# Dielectrophoretic estimation of resting membrane potential in excitable cells

**DOI:** 10.1371/journal.pone.0355723

**Published:** 2026-08-14

**Authors:** Matthew P. Johnson, Abdul Aziz Hamka, Stephanie Chacar, Okobi Ekpo, Moni Nader, Michael Pycraft Hughes

**Affiliations:** 1 Department of Biomedical Engineering and Biotechnology, Khalifa University of Science and Technology, Abu Dhabi, United Arab Emirates; 2 Department of Medical Sciences, Khalifa University of Science and Technology, Abu Dhabi, United Arab Emirates; 3 Department of Biological Sciences, Khalifa University of Science and Technology, Abu Dhabi, United Arab Emirates; 4 Department of Physiological Sciences, College of Medicine, Alfaisal University, Riyadh, Saudi Arabia; 5 Biotechnology Center, Khalifa University of Science and Technology, Abu Dhabi, United Arab Emirates; Lucian Blaga University of Sibiu: Universitatea Lucian Blaga din Sibiu, ROMANIA

## Abstract

The cell membrane potential (*V*_*m*_) is of paramount significance in cell electrophysiology, most notably in excitable cells found in muscle and nervous tissues. *V*_*m*_ is the voltage between the cell interior and extracellular space, and arises due to the diffusion potentials of potassium, chloride and sodium across the membrane. We recently reported a method to determine the resting membrane potential (RMP) of non-excitable cells including blood cells, chondrocytes, macrophages, and cancer cells, using a label-free, non-destructive, high-throughput method based on the electrical phenomenon *dielectrophoresis* (DEP). In this manuscript, we extend this technique to include the principal excitable cells, by altering the model to account for the different mechanisms which generate *V*_*m*_ in these cells. Our results indicate that unlike the RMP in non-excitable cells, excitable cells may have a smaller potential measured across the membrane itself, with sizeable extracellular component of *V*_*m*_ across the electrical double layer, measurable in the cell ζ-potential. When this was accounted for, the adapted model yielded results comparable to values in the literature. The model produced estimated values of RMP of −71.8 mV for SH-SY5Y neuroblastoma cells, and −74.2 mV in H9c2 cardiomyoblasts. Moreover, analysis of cardiomyoblasts in media with low concentrations of extracellular ions suggests that DEP can yield estimates of RMP in these media which align with published data for myocytes, with values of −54.1 mV between 100–600 mSm^-1^ and +12.7 mV below 100 mSm^-1^. This suggests that not only is DEP capable of accurate determination of *V*_*m*_ across the full range of cell types, but also that it could be used to examine ion channel behaviour and its effect on *V*_*m*_ across many ion concentrations.

## 1. Introduction

The resting membrane potential (RMP) of cells represents the “housekeeping” state of cellular electrophysiology; that is, the electrical condition in which cells remain in the absence of external stimuli which may alter the membrane potential (*V*_*m*_) [1]. Whilst many cells exhibit changes in *V*_*m*_ over periods of hours or days, such as circadian rhythmic potentials in red blood cells [2] or regulation of the cell cycle [3], the RMP is arguably most important in excitable cells. This class of cells, comprising muscle, nerve, and pancreatic islet β cells, relies on rapid (sub-millisecond) changes in *V*_*m*_ known as action potentials (APs) to conduct information in nerves or to regulate muscle contraction [4,5]. Understanding the intricate relationship between RMP and APs is central for designing pharmaceutical interventions to control cellular electrical activity [6].

Historically, excitable cells have been the primary vehicles through which we have understood how the sequestration and movement of ions play a role in cellular function, following early studies by Hodgkin and Katz [7], Cole and Curtis [8], and Goldman [9] in the early-to-mid 20^th^ century, and from which we derive the standard models subsequently applied to all cells. Using wire electrodes inserted into squid giant nerve cells, as well as by changing extracellular media and perfusing intracellular media, they developed the first models of cell electrophysiology. David Goldman developed the “constant field” model [9] which was later adapted by Hodgkin and Katz [7] to yield the “Goldman-Hodgkin-Katz” (GHK) equation, now widely adopted as the standard model of cell electrophysiology:


$$\begin{array}{c} V_{m}=\frac{RT}{F}ln\left(\frac{P_{\left({Na}^{+}\right)}{Na}_{med}^{+}+\;P_{\left(K^{+}\right)}K_{med}^{+}+P_{\left({Cl}^{-}\right)}{Cl}_{cyto}^{-}\;}{P_{\left({Na}^{+}\right)}{Na}_{cyto}^{+}+\;P_{\left(K^{+}\right)}K_{cyto}^{+}+P_{\left({Cl}^{-}\right)}{Cl}_{med}^{-}}\right) \end{array}$$ (1)

where *P*_*(x)*_ is the permeability for ion *x* (either Na^+^, Cl^-^ or K^+^) and represents measure of how easily a specific ion can cross the membrane; the subscripts *cyto* and *med* refer to ion concentrations of Na^+^, Cl^-^ or K^+^ in the cytoplasm or extracellular medium respectively; and *R*, *T* and *F* have their usual thermodynamic meanings. If one ion, such as K^+^ or Cl^-^ dominates, the expression reduces to the simpler, earlier form of the Nernst equation proposed by Bernstein [10]:


$$\begin{array}{c} V_{m}=\frac{RT}{F}\text{ln}\left(\frac{K_{med}^{+}}{K_{cyto}^{+}}\right) \end{array}$$ (2a)

or


$$\begin{array}{c} V_{m}=\frac{RT}{F}\text{ln}\left(\frac{{Cl}_{cyto}^{-}}{{Cl}_{med}^{-}}\right) \end{array}$$ (2b)

Whilst the AP was the most interesting aspect of excitable cells in the early years of electrophysiology research, the RMP is important across all the cells of the body; it is present in every cell, albeit at different voltages corresponding to different cellular functions. Smaller (depolarised, nearer to zero) RMP is associated with proliferating cells and cancer cells which tend to upregulate ion channels that facilitate cell proliferation and survival [11]. Ion channel modulation is an important aspect of pharmacological interventions, and consequently the measurement of RMP is a vital tool in cell biology and pharmacology [6]. However, measurement of *V*_*m*_ is difficult due to the inaccessibility of the cytoplasm; whilst direct measurement via either electrodes or saline-filled micropipettes using the patch-clamp method is possible, both methods are challenging and time consuming. Automated measures are available but tend to be expensive. Alternative methods are generally only able to produce indirect measures, allowing comparison of cells but not absolute values. The most common method is the measurement of fluorescence from *V*_*m*_-sensitive dyes, some of which can produce absolute measures via two-photon measurement [12–17]. However, these have limitations including issues with the biochemical interactions of cells and dye which could potentially cause shifts in ion gradients in these cells [18,19].

In our recent work [20], we demonstrated that dielectrophoresis (DEP) can provide a contactless, label-free method of directly sensing *V*_*m*_ in non-excitable cells. DEP is the induced motion of suspended particles in a non-uniform electric field. The effect is related to electrophoresis, a widely used tool in molecular biology for separations and protein identification. In DEP, the dielectric properties (conductance and capacitance) are dominant rather than surface charge [21], and the effect is present in both direct and alternating currents, allowing multiple frequencies to be used. The relationship between the frequency and the cell movement is given by the multi-shell Clausius-Mossotti factor [22], a mathematically robust model that is fitted to measurement of the induced motion of particles across a wide range of frequency. This model yields the conductivity and permittivity of the cytoplasm, and the capacitance and conductance of membranes. These properties have been shown to change as a function of frequency [20–24] in predictable ways, such as the relationship between cytoplasm and medium conductivities.

**Fig 1:**
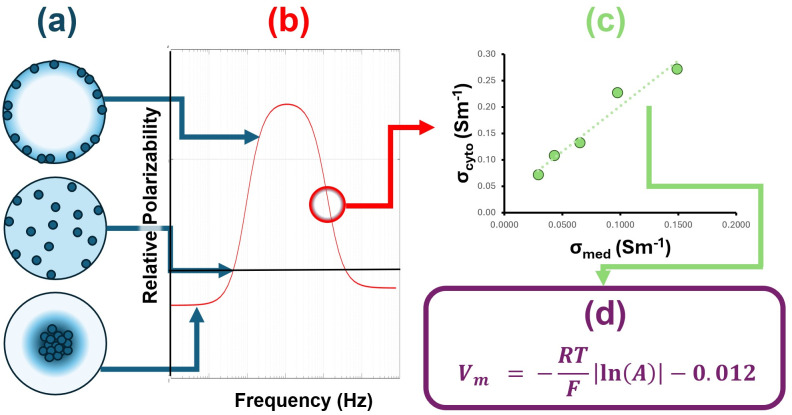
A schematic showing the process by which *V*_*m*_ is obtained by DEP data. (a) DEP is measured using electrodes to determine the electrical properties of cells. Electrodes induce attractive (top), neutral (middle) or repulsive (bottom) motion in cells with respect to the electrodes (large outer circles). (b) The DEP response of a population of cells is examined to determine the Clausius-Mossotti model of the cell. At high frequencies the dispersion frequency yields the cytoplasm conductivity *σ*_*cyto*_ for the experimental medium conductivity (*σ*_*med*_). (c) Measurements are taken at multiple values of *σ*_*med*_ to determine the slope *A* of *σ*_*cyto*_ against *σ*_*med*_ over a range of conductivities. (d) The value of A is used to calculate *V*_*m*_.


As shown schematically in Fig 1, the RMP is determined by measuring the rate of change in cytoplasm conductivity *σ*_*cyto*_ as a function of medium conductivity *σ*_*med*_, referred to here as parameter *A*:

$$\begin{array}{c} A=\frac{\delta \sigma_{cyto}}{\delta \sigma_{med}} \end{array}$$ (3)

This was processed using a variant of the Nernst equation; for slopes of A > 1, it was found that the expression


$$\begin{array}{c} V_{m}=\;\frac{RT}{F}\text{ln}\left(\frac{\text{1}\;}{A}\right)+\Psi_{offset}=\;\frac{RT}{F}\text{ln}\left(\frac{{\delta \sigma }_{med}\;}{{\delta \sigma }_{cyto}}\right)+\Psi_{offset} \end{array}$$ (4)

fitted, which is comparable to the Nernst equation for K^+^ in equation (2a). Similarly, where A < 1, the relationship was as follows:


$$\begin{array}{c} V_{m}=\;\frac{RT}{F}\text{ln}(A)+\Psi_{offset}=\;\frac{RT}{F}\text{ln}\left(\frac{{\delta \sigma }_{cyto}\;}{{\delta \sigma }_{med}}\right)+\Psi_{offset} \end{array}$$ (5)

This is analogous to the Nernst equation for Cl^-^ in equation (2b). In both cases, the *Ψ*_*offset*_ term has the same value of −0.012V and represents the cell’s surface potentials. Whilst the two behaviour suggest different physical processes may be present in these two conditions, equations (4) and (5) can be generalized into a single equation, thus:


$$\begin{array}{c} V_{m}\;=\;-\frac{RT}{F}\left|\text{ln}\left(\frac{{\delta \sigma }_{cyto}\;}{{\delta \sigma }_{med}}\right)\right|+\Psi_{offset} \end{array}$$ (6)

This expression was found to be applicable across all cells tested, including cancer cell lines such as HeLa and Jurkat, primary chondrocytes, RBCs, platelets, macrophages, and mesenchymal stem cells [20]. However, the model was not applied to excitable cells. The reasoning for this was that non-excitable cells do not require the maintenance of a highly polarised RMP to function. Since the DEP method involves measuring cells in media with substantially lower extracellular ion concentration than those in which cells normally live, one could suggest that this will result in an exchange of ions and a lower cytoplasm conductivity in line with the model in Equations (4)-(6). This is difficult to verify experimentally since patch clamp does not work in low ionic strength media, due to difficulties with seal formation. However, studies with RBCs using proton ionophores have shown that whilst these cells typically have an RMP of around -12mV (physiological strength), at conductivities equal or below 10% of physiological strength this RMP increased to around +20mV [21]. Conversely, it has been known for many decades that neurons maintain a *V*_*m*_ of around -70mV even when the extracellular ionic strength is reduced to 20% of the physiological level [7]. This is almost certainly due to the presence of a negative-feedback mechanism present in excitable cells to maintain a large RMP, because the mechanism for triggering APs is conventionally activated by the elevation of RMP above -55mV by the action of ligand-gated ion channels [1]. Since elevating the RMP above this point, for extended periods, would be detrimental to the cell (akin to tetany in muscle cells), a mechanism to maintain a polarised RMP is necessary and has been observed in the experiments recounted above.

In this study, we monitored the electrophysiological behaviour of excitable cells (neuroblastoma and cardiomyoblast cell lines) over a range of conductivities using DEP. We also determined the ζ-potential, the extracellular potential adjacent to the membrane and measured at the slipping plane, which has been shown to contain a component related to *V*_*m*_. Our results suggest that excitable cells differ from their non-excitable counterparts, with Equation (6) fitting following application of a correction factor. The assumption from which we derived a model that was further validated using ζ-potential measurement. The results suggest that excitable cells tend to behave in a similar manner to non-excitable cells but may be adapted with a feedback mechanism to regulate RMP. Furthermore, the results obtained in H9c2 cardiomyoblasts also suggest that DEP can be used to measure *V*_*m*_ across the conductivity range, offering a useful alternative to patch clamp for studying ion channels.

## 2. Materials and methods

### 2.1. Cell preparation

H9c2 cardiomyoblasts (ATCC CRL-1446) were cultured in T75/T25 flasks with DMEM (Gibco Life Sciences, 11965−092) supplemented with 10% FBS (Gibco Life Sciences, A47668-01) and 1% Penicillin Streptomycin (Gibco Life Sciences, Cat:15140–122). SH-SY5Y neuroblastoma cells (ATCC CRL-2266) differentiated to a neuron-like phenotype [25,26] were cultured in T25 flasks with 1:1 EMEM/F12 growth media (AddexBio, C0005-02), 10% FBS, and 1% Penicillin-streptomycin. Both cell types were cultured in 37˚C incubators with 5% CO_2_.

When 70–90% confluent, the cells were washed and detached with 0.25% (w/v) Trypsin-EDTA (Sigma Aldrich, T4049). After suspension, complete medium was added to inactivate the trypsin, and the solution was centrifuged at 700 RCF for 5 minutes. The supernatant was removed, and cells were resuspended in DEP media (248 mM Sucrose, 16.7 mM D-Glucose, 250 μM MgCl_2_, 100 μM CaCl_2_, and adjusted to 43 mS/m conductivity with Dulbecco’s Phosphate Buffered Saline (GE Life sciences, South Logan, Utah, Magnesium- and Calcium-free). Cells were centrifuged again at the same conditions before resuspending in 1.2 mL of DEP media solutions with a range of different conductivities. Conductivities used ranged from 43 to 750 mS/m, prepared by adding further DPBS to the stock solution whilst measuring with a conductivity meter. Cell concentrations were approximately 1x10^6^ cells per mL, measured using an Invitrogen Countess 3 automated cell counter (Thermo Fisher, USA) after sample analysis, which also provided cell diameters for Clausius-Mossotti DEP fitting, and post-experimental viabilities of average 82.5% with standard error of 2.0% by the standard Trypan Blue test. We performed at least 12 biological replicates for each cell type.

### 2.2. Dielectrophoresis recordings

The 3DEP system (DEPtech Hastings, UK) [27] simultaneously measured populations of 10–20,000 cells, producing complete DEP spectra in approximately 30 seconds, per conductivity. The 3DEP platform uses 3D chips in which electrodes form stripes around the circumference of well-shaped structures approximately 1 mm in diameter and 1 mm high. Within these wells, cells respond with either positive (attractive) DEP or negative (repulsive) DEP, moving either towards the electrodes or towards the centre of the well. These are observed using a camera and telecentric optic, allowing the measurement of speed and direction of net cell movement, as shown schematically in Fig 1a. The system uses 20 wells, each receiving a different frequency, allowing complete DEP spectra to be acquired simultaneously, as shown in Fig 1b; *σ*_*cyto*_ was then determined using a mathematical model [22] to measure the higher dielectric dispersion, indicated by the circle in Fig 1b. Following our previous work, we investigated *A* from equation (3) (Fig 1c), which was then used in Equation (6) (Fig 1d).

For each measurement, 400 µL per sample was used to take 3–6 complete DEP spectra. DEP analysis was performed using a 3DEP cytometer (DEPtech, Uckfield UK) [27]. For each repeat, the 3DEP applied 10Vpk for 30 seconds across 20 frequencies from 10kHz to 65MHz simultaneously. The 3–6 repeats were averaged for each frequency and single-shelled Clausius-Mossotti models were applied with the autofitting script by Tsai et al. [28] to extract cytoplasm conductivity *σ_cyto_*. *V*_*m*_ was estimated from *σ_cyto_* gradients against medium conductivity as outlined previously [20]. Data were analysed post-collection using GraphPad Prism 10 (GraphPad, Boston, USA).

### 2.3. ζ-potential measurement

The ζ-potential was measured simultaneously with DEP analysis. For each sample, mean ζ-potential and experimental conductivity were measured using a Malvern Panalytical Zetasizer Lab Series Blue (Malvern, UK). A volume of 800 µL of undiluted cell sample was inserted into disposable DTS1070 cuvettes (Malvern, UK). The ζ-potential was recorded 5 times per sample. Data analysis was performed using GraphPad Prism 10 (GraphPad, Boston, USA).

## 3. Results and discussion

### 3.1. Estimation of *V*_*m*_ using DEP

**Fig 2:**
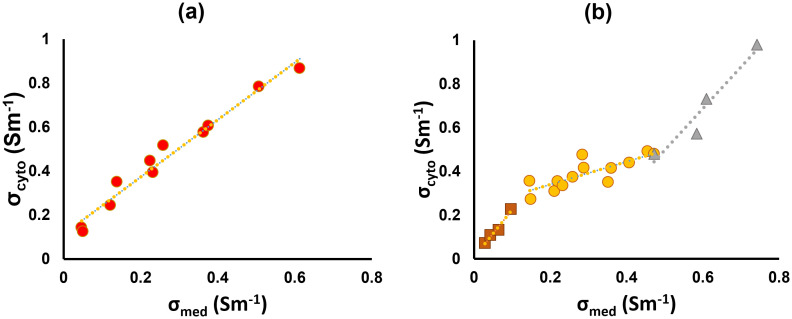
DEP-derived values of cytoplasm conductivity *σ*_*cyto*_ as a function of medium conductivity σ_med_, acquired for (a) SH-SY5Y cells and (b) cardiomyoblasts. Broken lines indicate best linear fits; for (b), data were collected into three slopes to maximise R^2^, with different colours and shapes used to differentiate between the groups. The different groups suggest that the slope is augmented by *V*_*m*_, which varies in value across the conductivity range.

Both cell types produced DEP spectra across the wide conductivity range examined here. From these spectra, the mean value of *σ*_*cyto*_ was obtained for each biological replicate, which was plotted against the medium conductivity *σ*_*med*_ at which the measurement was taken, as shown in Fig 2a (SH-S5Y5 neuroblastoma cells) and Fig 2b (H9c2 cardiomyoblasts). Considering neuroblastoma cells first, the data followed a similar trend to other cells [20], with a monotonic linear increase in *σ*_*cyto*_ as a function of *σ*_*med*_. When the best-fit slope of the graph was determined, it was found that the value of *A* from Equation (3) was measured at 1.30 (r^2^ = 0.97) between 50 mSm^-1^ and over 750 mSm^-1^. Cardiomyoblasts exhibited a different pattern, with multiple slopes connected by two distinct inflection (“pivot”) points. When we attempted to determine best-fit lines using linear regression, we found that below 100 mS/m the data showed a strong correlation (R^2^ = 0.94) with a line where *A* = 1.29; between 100mSm^-1^ and around 600 mSm^-1^ the slope was *A* = 0.65 (R^2^ = 0.80), whilst above that the data showed a similarly strong correlation (r^2^ = 0.97) where *A* = 1.90.

When we used these data in Equation (6), we obtained estimated values of *V*_*m*_ of −18.6 mV for neuroblastoma cells, whilst for cardiomyoblasts we obtained values of −18.4 mV at low conductivity, −22.9 mV between 100−600 mSm^-1^, and −28.2 mV above this. These values are substantially lower than the recorded values for these cells using microelectrodes and patch clamp, which estimate typical values of *V*_*m*_ in physiological media of around −70 mV [1]. Our predicted values reflect depolarization with respect to the threshold above which the cells begin AP production, which for both cell types is approximately -55mV. Clearly, the predicted values from Equation (6) are substantially less negative when compared to values measured by other methods. To account for this, we considered the distribution of *V*_*m*_ between cytoplasm and bulk medium.

It has been observed in a range of cells including blood cells [21,23], cancer cells [29] slime molds [30] and yeast cells [31], that the extracellular potential (commonly measured as the ζ-potential at the hydrodynamic plane of shear) contains two components; one derives from the potential arising from the surface charge, whilst a second is a fraction of the membrane potential. In experiments with blood cells [21] and yeast cells [31] this has been estimated to be around 0.37 *V*_*m*_ The mechanism for this has yet to be elucidated; it is far larger than would be anticipated for simple capacitive potential, for example. However, it has clearly been observed in dynamic changes of *V*_*m*_ such as red cell depolarization [21] and action potentials in cardiomyoblasts [32]. This proportion of *V*_*m*_ at the outer surface was given the symbol *Ξ* [21].

If *Ξ V*_*m*_ is dropped across the extracellular electrical double layer, it is reasonable to expect that a similar potential will be dropped across the intracellular double layer between the inner membrane surface and the bulk cytoplasm. If this is the case, then remaining portion of *V*_*m*_ that is dropped across the membrane itself is *V*_*m*_/(1–2*Ξ*). If we add this to Equation (6), we obtain the expression


$$\begin{array}{c} V_{m}\;=\;-\frac{\text{1}}{\left(\text{1}-\text{2}\Xi \right)}\left(\frac{RT}{F}\left|\text{ln}(A)\right|\right)+\Psi_{offset} \end{array}$$ (7)

When we re-analysed the gradient data with this adapted model, we obtained measurements which were much closer to those measured by conventional electrophysiological methods. Considering the SH-SY5Y cells first, we found that equation (7) yielded *V*_*m*_ = 71.8 mV for SH-SY5Y cells across the conductivity range; this aligns closely with reported values of *V*_*m*_ in neurons, which typically fall in a range between −70 mV and −75 mV [32]. When we applied Equation (7) to the cardiomyoblast data, we found that it yielded three values; below a medium conductivity of 100 mSm^-1^ it estimated *V*_*m*_ = −36.7 mV (r^2^ = 0.97); −54.1 mV between 100–600 mSm^-1^ (r^2^ = 0.65); and −74.2 mV above this (r^2^ = 0.93). However, we can also refine this further. Examination of the relationship between *V*_*m*_ and ζ-potential in *Candida albicans* [31] at low medium conductivities suggests that in this regime, *Ξ* is negative (taking the value of −0.35); since it is not possible to have a potential divider that reverses polarity, the implication that *V*_*m*_ is in fact positive which, since Equation (7) contains a modulus operator, we are unable to identify from determining *A*. Similarly, direct measurements of *V*_*m*_ (validated using ζ-potential) in red blood cells at around 140 mSm^-1^ medium conductivity and below, also shows that *V*_*m*_ is around +20 mV under these conditions [21], whilst Hodgkin and Horowicz [33] reported values up to +4 mV in low-Na^+^, low-Cl^-^media. If we adapt Equation (7) to account for this by removing the negative multiplier from the start of the expression, then the estimated value of *V*_*m*_ for the lowest conductivity band is + 12.7 mV.

We then compared our *V*_*m*_ estimation as a function of medium conductivity to published electrophysiology data. For our myoblast data, we compared DEP analysis to recordings of cells from the sartorius muscle of the frog published by Hodgkin and Horowicz [33], whilst we compared the data of the neuroblastoma cells to Hodgkin and Katz’ work on giant squid neurons [7]. In both cases, recordings were taken as a function of extracellular ion concentration, allowing direct comparison to our work. Considering neuroblastoma cells first, Hodgkin at Katz [7] reported that the effect of variation of extracellular ion concentration (at six conductivities from 20% to 159% of physiological strength) had little impact on the RMP, which was observed to vary by an average of ±4mV at 20% of physiological strength and no more than ±2 mV at any other concentration. Whilst the average value reported by Hodgkin and Katz was around −48 mV [7], mammalian neuron RMP is widely reported to be around −70 mV [34], in close agreement to our estimate.

**Fig 3:**
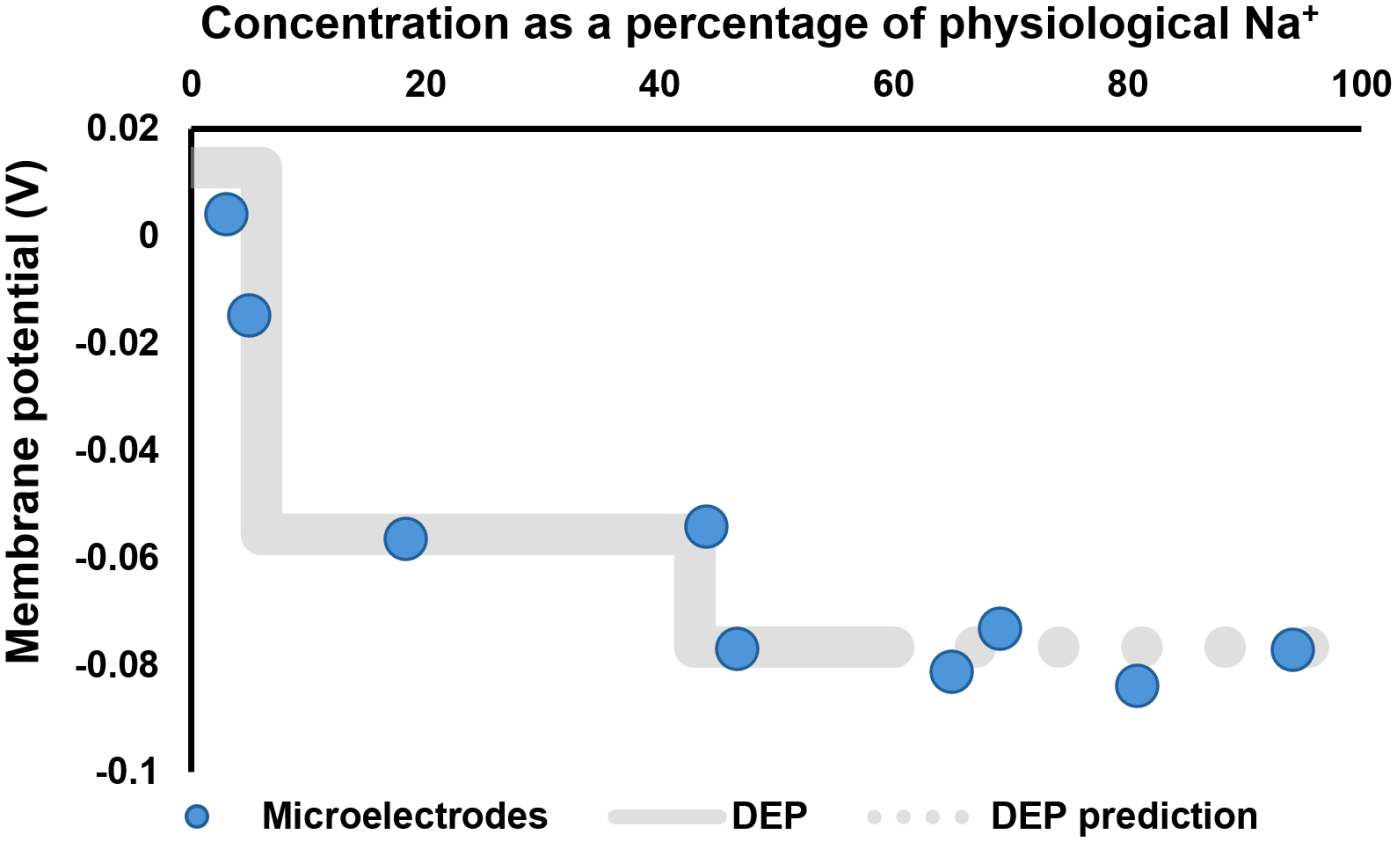
A comparison of the DEP-derived *V*_*m*_ using equation (7) and the slopes of Fig 2b (grey line) compared to published data acquired using microelectrodes from frog myocytes [33] (blue points). The dotted grey line extrapolates the value of Vm acquired at higher conductivity; comparison to published data suggests that the value acquired in the higher conductivity region is likely representative of the value under physiological conditions.

In our analysis of cardiomyoblasts, we reported three different values of RMP at different conductivities. When this was compared to the experimental data of Hodgkin and Horowicz [33], we found that they had observed similar behaviour at different extracellular concentrations (we calibrated this for Na^+^ concentration, the dominant cation in both their medium and ours, and the one most closely examined in the literature), as shown in Fig 3. In both the published data and ours, three plateaux were observed, with a high degree of correlation between results taken by both methods. Our highest measurement was at 0.77 Sm^-1^, the prediction of our system is in line with previous work, that the estimated value of *V*_*m*_ in physiological media is similar to this (as indicated by the dotted line in the figure).

### 3.2. ζ-potential measurement of excitable cells

**Fig 4:**
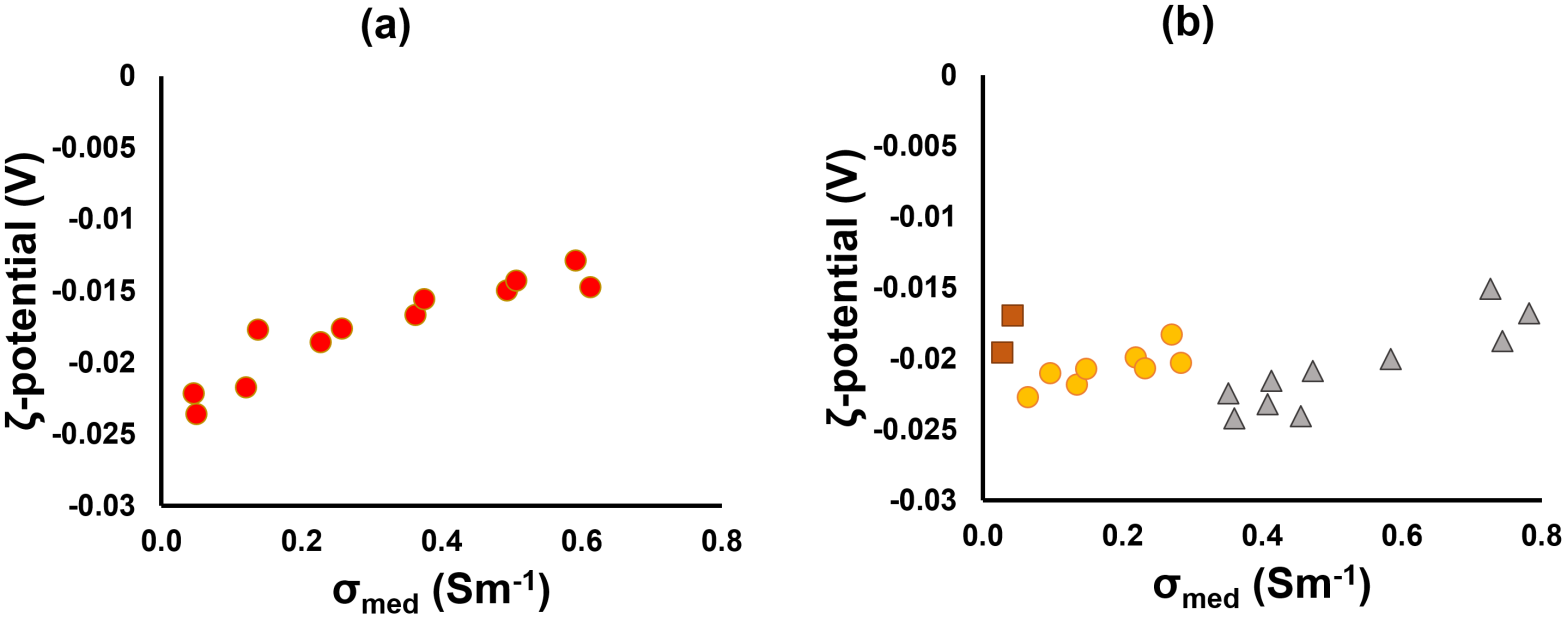
*ζ*-potential as a function of medium conductivity σ_med_, acquired for (a) the SH-SY5Y cells and (b) cardiomyoblasts. For (b), data were collected into three slopes to maximise R^2^, with different colours and shapes used to differentiate between the groups. The different groups suggest that ζ-potential is augmented by *V*_*m*_, which varies in value across the conductivity range.

In addition to DEP, we also measured the ζ-potential of cells across a wide range of conductivities. These are shown in Fig 4a for neuroblastoma cells and Fig 4b for cardiomyoblasts. As with the DEP results, the ζ-potential of the SH-SY5Y cells followed the anticipated pattern [21] for a negative exponential trend towards depolarization, though the trend suggested that the depolarization was asymptotic towards −10 mV rather than 0 mV. Conversely, cardiomyoblasts remained relatively constant across conductivities, producing values −21.3 ± 2.0 mV throughout the range, and trended slightly higher with higher conductivity. However, closer examination of the cardiomyoblasts data suggested that ζ-potential are actually reduced with increasing conductivity but is subject to successive stages of negative displacement.

To better understand this, we considered the origin of the ζ-potential. This describes the electrical potential arising due to the surface potential *Ψ*_*surface*_ of a suspended solid, but diminishing with increasing distance due to the screening effect of the electrical double layer. The ζ-potential itself corresponds to the potential at the hydrodynamic plane of shear, a distance *x* from the surface, at which the surrounding solution ceases to be hydrodynamically stagnant. It can be calculated using the expression [35]


$$\begin{array}{c} \zeta =\Psi_{surface}e^{-\kappa x} \end{array}$$ (8)

Where *κ* is the reciprocal of the Debye length (that is, the effective thickness of the double layer) given by the expression [35]


$$\begin{array}{c} \kappa =\sqrt{\left(\frac{\text{2}czF^{\text{2}}}{\varepsilon RT}\right)} \end{array}$$ (9)

where *z* is the counterion valency, *c* the electrolyte concentration (mol m^-3^), *ε* the electrical permittivity of the double layer and *R, T* and *F* are as before. As can be seen, the relationship between *κ* and *c*^½^ in Equation (8) suggests that *ζ* should decrease with increasing *c*. However, whilst this model describes inanimate objects well, cells have exhibited an additional component due to the membrane potential [21,23,29–31]. Hughes et al. [21] suggested that the ζ-potential of RBCs is altered by *V*_*m*_ thus:


$$\begin{array}{c} \zeta =\zeta^{\prime }+\Xi V_{m} \end{array}$$ (10)

Where ζ is the measured ζ-potential, ζ’ is the ζ-potential due solely to the surface charge, and Ξ is the proportion of membrane potential that is observed in the ζ-potential [21]. If we combine equations (8) and (10), we find that


$$\begin{array}{c} \zeta =\left(\Xi V_{m}\right)e^{-\kappa x}+\;\zeta^{\prime } \end{array}$$ (11)

**Fig 5:**
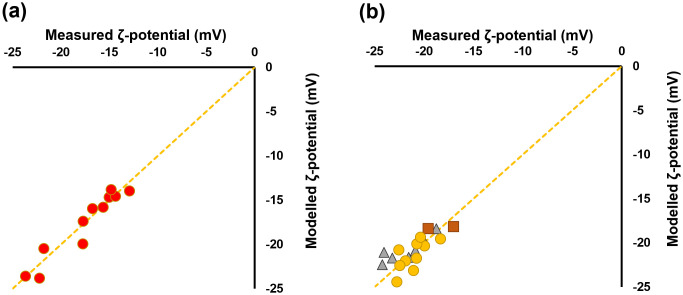
Comparison of the measured value of ζ-potential with the expression described in Equation (11). SH-SY5Y cells (a) yielded a constant value of *V*_*m*_ for all values of ζ-potential, whereas cardiomyoblasts (b) yielded different values in line with the groupings in Fig 3b.

We used this to fit the observed behaviour of the cells. Applying this first to the ζ-potential of the SH-SY5Y cells, these showed a simple exponential drift towards an offset value. This could easily be fitted to Equation (11) using least-squares regression; the greatest value of r^2^ (0.83) was obtained when *ΞV*_*m*_ had a value of 22.9 mV, ζ’ had a value of −8.5 mV, and *x* was 0.2 nm from the surface. If we assume *Ξ* = 0.37, then this yields a value of *V*_*m*_ of 62 mV; conversely, assuming *V*_*m*_ = -70mV yields *Ξ* of 0.33. This parameter set yielded the correspondence shown in Fig 5a. When the same approach was applied to cardiomyoblasts, it was necessary to apply different values of *V*_*m*_ for different conductivity ranges. A best fit was identified when *ΞV*_*m*_ had values of −4.7 mV at conductivities below 60 mSm^-1^, −20 mV between 60–350 mSm^-1^ and −32 mV above this (r^2^ = 0.98). The value of ζ’ was found to fit well when −11.5 mV was used throughout. This yielded the correspondence shown in Fig 5b. For the lower two values this yielded 0.37 exactly for the reported values of *V*_*m*_, whilst at higher conductivities it suggested that *V*_*m*_ = 86.5 mV.

There are two important implications arising from this data; firstly, it suggests that there is a significant component of *V*_*m*_ arising at the slip plane and altering ζ-potential, and secondly it further validates the observed changes in the value of *V*_*m*_ across the conductivity range. This suggests that the previous estimates of *Ξ* = 0.37 arising at the surface that is assumed in Equation (7), though the difference between this value (observed here for cardiomyocytes) and the value of 0.33 observed for neurons suggests two possibilities; that there is a range of values for different kinds of cells, or that the parameter is the same for all cells, but that there is a degree of variability in the estimation. Further analysis is required in order to better understand the origin of the effect before this can be resolved.

### 3.3. Pivot behaviour in cardiomyoblasts suggests DEP can measure ion channel activity

The variation in *A* across the conductivity range between the SH-SY5Y cells and cardiomyoblasts showed one important difference; for neuroblastoma cells, parameter *A* was constant, and greater than 1 throughout the conductivity range, suggesting that resting *V*_*m*_ remains constant for a wide range of extracellular composition, in agreement with physiological measurements taken by Hodgkin and Katz [7]. Conversely, cardiomyoblasts showed several values of *A*; this was above 1 at low conductivity, but became less than 1 at mid-range conductivities, and above 1 again at high conductivities. Comparison to the microelectrode-derived data of Hodgkin and Horowicz [33] showed again that the DEP model accurately represents observed behaviour in myocytes, with their measurements indicating the presence of three plateaux across the extracellular concentration range.

This difference in behaviour may lie with chloride channels such as ClC channels [36]. These are inwardly-rectified and voltage-gated at around −55 mV [36], which play a significant role in the RMP of skeletal muscles by preventing K^+^ accumulation at the T-tubules from propagating along the sarcolemma, avoiding unwanted spontaneous depolarizations [37]. In mammalian cardiac myocytes, Cl^-^ channels are also essential for regulating action potential duration and maintaining RMP under physiological conditions [38–40]. Conversely, neuronal cells lack significant ClC channel expression [36]; instead, these cells moderate RMP through increased expression of voltage-gated potassium channels (K_v_) which are key regulators of many neurophysiological functions including RMP, neuronal membrane excitability, spontaneous firing rate, channels kinetics, cell proliferation, and apoptosis [41,42]. The estimated value of RMP in cardiomyoblasts is held at around −55 mV from around 10% of physiological ion strength (around 14 mM extracellular Cl^-^) to around 50%. This may suggest that the ClC channels are primarily responsible for setting the RMP in low ionic strength media, with *V*_*m*_ set to the channel activation potential until extracellular K^+^ is sufficient to enable higher potentials to be generated. This also suggests that *V*_*m*_ estimation by DEP may have potential applications in measurement of ion channel activity, and even for estimation of intracellular ion concentration with further development.

### 3.4. “Open loop” vs “closed loop” regulation?

The work also raises an interesting question regarding the nature of RMP generation between excitable and non-excitable cells. Equation (6) has been shown to be effective across a wide range of cells, including red blood cells, platelets, both suspension and adherent cancer lines, stem cells, primary chondrocytes, monocytes, with more cell types being analysed regularly. To date, no non-excitable cell has generated a result which this equation does not predict a value of *V*_*m*_ with reasonable accuracy (typically no more than ±2 mV variation) when compared to patch clamp data. Conversely, the two cell types here required an adjustment of 1/(1–2Ξ) to produce an accurate result. The need for this factor suggests a difference in the way the RMP is generated between excitable and non-excitable cells. Specifically, if the DEP-generated value of *V*_*m*_ indicates the value of electrical potential dropped across the membrane itself, then Equation (6) suggests that the potential is fully dropped across the membrane, whilst Equation (7) suggests that only a portion of *V*_*m*_ is found here, with the other portions being found in the adjoining inner and outer double layers.

This may point to differences in the regulation of intracellular ions. For most cells, maintenance of RMP is important, but in the absence of functional transients such as action potentials, the cell may be able to function in a wide range of extracellular ionic strengths. Conversely, regulation of both RMP and AP are fundamental to muscle and nerve function. This may require a mechanism that alters the potential to maintain intracellular ion concentration or alters intracellular concentration to maintain potential.

In electronics, the mechanism by which such values are maintained is through a feedback loop; systems with feedback (“closed-loop”) are much less susceptible to perturbations than those without a feedback mechanism (“open-loop”) [43]. In an open-loop controller, the output is dictated entirely by the difference between inputs; in a closed-loop controller, feedback is used such that the output regulates the inputs, resulting in a smaller controlling voltage that remains stable even when conditions change. These results suggest that excitable cells may use regulation of *V*_*m*_ across the membrane itself as a mechanism to control the potential between cytoplasm and bulk medium. Conversely, non-excitable cells may lack such a stabilising mechanism so the cytoplasm content is able to vary far more widely as extracellular conditions change, yielding substantially larger values of the coefficient *A* relating intracellular and extracellular ionic strength. Alternatively, the mechanism may allow cells to generate substantial values of *V*_*m*_ (which are typically hyperpolarised in excitable cells compared to non-excitable cells) without requiring commensurately large intracellular ion concentrations.

The precise mechanism for this feedback loop, and its functional relevance in excitable cells, are yet to be elucidated. However, the present data (generated by both DEP and ζ-potential) suggests that the mechanism, particularly the presence of over 30% of *V*_*m*_ at the extracellular surface, are both extant phenomena. Given that the surface concentration *c*_*surf*_ of ion *i* with valency *z*_*i*_ at a charged surface with potential *Ψ*_*o*_ with respect to that in the bulk medium *c*_*bulk*_ is governed by the Poisson-Boltzmann equation


$$\begin{array}{c} c_{surf}=c_{bulk}\text{exp}\left(\frac{{-z}_{i}e\Psi_{\text{0}}}{kT}\right) \end{array}$$ (12)

Where *k*, *T* and *e* have their usual thermodynamic meanings. Consider what happens if we augment *Ψ*_*o*_ with a portion of the membrane potential; that is, replace *Ψ*_*0*_ with *Ψ*_*0*_ + *ΞV*_*m*_. Since *V*_*m*_ is (from Equation (1)) dependent on the ion concentration at the surface, but the surface concentration (from equation (12)) is dependent on *V*_*m*_, this interdependence may form a feedback mechanism. Since feedback loops are commonly associated with maintaining stability, this suggests that closed-loop feedback is often associated with maintenance of stability, that may have possible role in the maintenance of a stable RMP across varying external ion concentrations.

## 4. Conclusion

We have described for the first time a robust method for the determination of *V*_*m*_ across excitable cells, that complements our previous non-excitable work, by using an adaptation that accounts for the difference between equilibrium and non-equilibrium sources of RMP. The results from DEP, confirmed by ζ-potential measurement, strongly suggest that while the RMP of non-excitable cells is due to unregulated internal cellular conditions, in excitable cells the requirement for RMP stability for avoidance of spontaneous firing means that RMP is maintained using a “closed loop” system with some form of feedback. By introducing a correction factor for excitable cells, we have extended our previous model to provide a simple, low-cost, label-free and robust method of measurement of absolute values of RMP across both excitable and non-excitable cells.

## Supporting information

S1 File The experimental data set used in this paper. (DOCX)
