# Supplementary material for: Dielectrophoretic estimation of resting membrane potential in excitable cells
Source: PLoS One. 2026 Aug 14;21(8):e0355723. doi: 10.1371/journal.pone.0355723 (PMC13475854; doi:10.1371/journal.pone.0355723)
Supplement: Supplementary file 1 [file pone.0355723.s001.docx]

| Sample conductivity (mS/m) | Cytoplasmic conductivity (S/m) | Zeta potential (mV) | | | | | | |
| --- | --- | --- | --- | --- | --- | --- | --- | --- |
|  |  | Avg | SD | Rep1 | Rep2 | Rep3 | Rep4 | Rep5 |
| **H9c2** | | | | | | | | |
| 28.89 | 0.0710 | -19.59 | 0.55 | -20.04 | -20.34 | -19.17 | -18.83 | -19.55 |
| 43.49 | 0.1080 | -17.01 | 3.29 | -12.01 | -20.32 | -16 | -20.94 | -15.77 |
| 65.11 | 0.1316 | -22.73 | 1.45 | -21.94 | -22.56 | -25.57 | -21.99 | -21.61 |
| 97.51 | 0.2266 | -21.06 | 0.44 | -21.82 | -20.8 | -21.23 | -20.55 | -20.91 |
| 135.6 | 0.3555 | -21.85 | 1.02 | -20.1 | -22.81 | -21.51 | -21.94 | -22.9 |
| 148.6 | 0.2718 | -20.76 | 0.47 | -20 | -20.86 | -20.51 | -21.14 | -21.3 |
| 211 | 0.3084 | -22.55 | 0.32 | -22.21 | -22.98 | -22.86 | -22.22 | -22.48 |
| 219.4 | 0.3548 | -19.95 | 0.51 | -19.17 | -19.5 | -20.3 | -20.33 | -20.44 |
| 232.6 | 0.3347 | -20.71 | 0.32 | -20.34 | -20.72 | -20.74 | -20.49 | -21.28 |
| 259.2 | 0.3740 | -22.57 | 0.58 | -21.84 | -22.17 | -22.44 | -22.94 | -23.48 |
| 271 | 0.4152 | -18.34 | 2.81 | -18.63 | -14.37 | -18.39 | -17.25 | -23.07 |
| 284.5 | 0.4762 | -20.35 | 0.42 | -21.04 | -20.49 | -20.37 | -20 | -19.83 |
| 351.4 | 0.3504 | -22.45 | 1.44 | -21.17 | -21.45 | -21.43 | -23.3 | -24.89 |
| 359.9 | 0.4144 | -24.21 | 1.47 | -22.34 | -22.57 | -24.86 | -25.41 | -25.88 |
| 407.6 | * | -23.21 | 1.59 | -20.56 | -22.95 | -22.88 | -24.47 | -25.19 |
| 412.9 | 0.4397 | -21.55 | 1.88 | -19.26 | -20.39 | -20.61 | -23.18 | -24.29 |
| 455.3 | 0.4912 | -24.05 | 1.58 | -21.33 | -23.28 | -24.92 | -25.7 | -25.04 |
| 472.1 | 0.4800 | -20.87 | 1.44 | -20.58 | -18.34 | -21.36 | -22.72 | -21.36 |
| 584.1 | 0.5718 | -20.04 | 1.11 | -21.86 | -19.85 | -19.71 | -20.37 | -18.43 |
| 726.4 | 0.7319 | -15.07 | 0.71 | -13.89 | -15.35 | -16.04 | -15.22 | -14.84 |
| 743.6 | * | -18.77 | 0.71 | -18.15 | -18.52 | -18.07 | -19.97 | -19.15 |
| 782.2 | 0.8212 | -16.79 | 0.59 | -15.88 | -16.35 | -17 | -17.47 | -17.26 |
|  |  |  |  |  |  |  |  |  |
| **SH-SY5Y** | | | | | | | | |
| 46.87 | 0.1430 | -22.20 | 1.15 | -20.93 | -20.72 | -22.89 | -23.58 | -22.87 |
| 50.37 | 0.1252 | -23.64 | 0.93 | -23.09 | -22.67 | -23.14 | -23.98 | -25.3 |
| 121.4 | 0.2447 | -21.75 | 1.64 | -24.94 | -20.87 | -21.34 | -20.29 | -21.31 |
| 138.2 | 0.3507 | -17.72 | 1.57 | -19.61 | -19.28 | -17.62 | -15.41 | -16.7 |
| 224.1 | 0.4471 | -13.88 | 0.71 | -14.57 | -14.55 | -12.86 | -13.2 | -14.21 |
| 231.7 | 0.3941 | -23.34 | 2.17 | -20.98 | -21.93 | -27.31 | -22.9 | -23.57 |
| 258.7 | 0.5166 | -17.68 | 0.68 | -18.95 | -17.77 | -17.06 | -17.4 | -17.23 |
| 363 | 0.5767 | -16.73 | 0.62 | -15.65 | -17.32 | -17.33 | -16.53 | -16.82 |
| 375.6 | 0.6061 | -15.62 | 0.71 | -14.68 | -15.56 | -16.31 | -16.52 | -15.05 |
| 494.4 | 0.5960 | -14.99 | 0.32 | -15.17 | -15.25 | -14.88 | -15.24 | -14.41 |
| 507.1 | 0.7841 | -14.34 | 0.35 | -14.43 | -13.69 | -14.71 | -14.35 | -14.52 |
| 591.9 | 0.7820 | -12.91 | 1.54 | -11.68 | -11.99 | -12.56 | -12.4 | -15.92 |
| 612.9 | 0.8671 | -14.78 | 1.12 | -14.18 | -15.04 | -13.53 | -14.36 | -16.81 |

* 3DEP results did not generate clean DEP spectra for cytoplasmic conductivity to be extracted. Zeta potential, as measured independently by the Malvern Zetasizer was still included.
